# 伴TET2双等位基因失活的骨髓增生异常肿瘤患者分子学和临床特征及预后研究

**DOI:** 10.3760/cma.j.cn121090-20260115-00025

**Published:** 2026-04

**Authors:** 昕 王, 琳琳 刘, 冰 李, 玉娇 贾, 承文 李, 铁军 秦, 泽锋 徐, 士强 曲, 丽娟 潘, 清妍 高, 蒙 焦, 志坚 肖

**Affiliations:** 1 中国医学科学院血液病医院（中国医学科学院血液学研究所），血液与健康全国重点实验室，国家血液系统疾病临床医学研究中心，细胞生态海河实验室，天津 300020 State Key Laboratory of Experimental Hematology, National Clinical Research Center for Blood Diseases, Haihe Laboratory of Cell Ecosystem, Institute of Hematology & Blood Diseases Hospital, Chinese Academy of Medical Sciences & Peking Union Medical College, Tianjin 300020, China; 2 天津医学健康研究院，天津 301600 Tianjin Institutes of Health Science, Tianjin 301600, China

**Keywords:** 骨髓增生异常肿瘤, TET2基因, 突变, 预后, IPSS-M, Myelodysplastic neoplasms, TET2 gene, Mutation, Prognosis, IPSS-M

## Abstract

**目的:**

探究伴TET2双等位基因失活的骨髓增生异常肿瘤（MDS）患者的临床特征及预后意义。

**方法:**

收集2016年8月至2025年6月就诊于中国医学科学院血液病医院且有二代基因测序数据的1 730例初诊原发MDS患者的病例资料，回顾性分析伴TET2双等位基因失活患者的临床特征及预后。

**结果:**

39例（2.3％）患者伴TET2双等位基因失活，其中有2例检出染色体4q24位点拷贝数中性杂合性缺失。与TET2单等位基因突变患者（147例）和无TET2基因突变患者（1 544例）相比，TET2双等位基因失活患者携带的基因突变更多［4（*IQR*：3，6）个对4（*IQR*：2，5）个对2（*IQR*：1，4）个，*P*<0.001］，合并ASXL1（38.5％对29.9％对20.5％，*P*＝0.001）、SRSF2（28.2％对12.9％对4.5％，*P*<0.001）、SF3B1（25.6％对18.4％对11.7％，*P*＝0.004）、EZH2（18.0％对7.5％对4.5％，*P*＝0.002）、CUX1（12.8％对3.4％对1.4％，*P*<0.001）、KRAS（10.3％对4.3％对2.1％，*P*＝0.006）、CBL（10.3％对3.4％对2.6％，*P*＝0.030）、ZRSR2（10.3％对6.1％对3.2％，*P*＝0.017）、CEBPA（7.7％对1.4％对1.2％，*P*＝0.019）基因突变比例更高，中位发病年龄更高［65（*IQR*：58，69）岁对61（*IQR*：50，67）岁对55（*IQR*：44，64）岁，*P*<0.001］，正常染色体核型比例也更高（69.2％对46.3％对44.8％，*P*＝0.024）。生存分析显示，伴TET2双等位基因失活患者中位总生存（OS）期为18.5（95％ *CI*：13.1～25.0）个月，较TET2单等位基因突变患者［40.6（95％ *CI*：22.8～60.9）个月］和无TET2基因突变患者［54.7（95％ *CI*：47.1～69.4）个月，*P*＝0.009］明显缩短。进一步按分子国际预后评分系统（IPSS-M）预后风险分层分析发现，在IPSS-M相对高危（IPSS-M较高危、高危、极高危）患者中，TET2双等位基因失活患者中位OS期为12.4（95％ *CI*：8.4～NA）个月，显著短于TET2单等位基因突变患者［19.7（95％ *CI*：14.9～39.9）个月］及无TET2基因突变患者［32.0（95％ *CI*：27.6～37.4）个月，*P*<0.001］；而在IPSS-M相对低危（IPSS-M极低危、低危、较低危）患者中未观察到显著差异。

**结论:**

伴TET2双等位基因失活的MDS患者具有特定的临床和分子学特征，在IPSS-M相对高危患者中预后较TET2单等位基因突变患者更差。

## Abstract (en)

**Objective:**

To explore the clinical characteristics and prognostic significance of myelodysplastic neoplasms（MDS）with biallelic TET2 inactivation（bi-TET2）.

**Methods:**

Clinical data from 1 730 newly diagnosed *de novo* MDS patients treated at Institute of Hematology & Blood Diseases Hospital, Chinese Academy of Medical Science & Peking Union Medical College between August 2016 and June 2025, with next-generation sequencing data, were retrospectively collected. The clinical characteristics and prognosis of patients with biallelic TET2 inactivation were analyzed.

**Results:**

Thirty-nine patients（2.3％）harbored bi-TET2, including two with copy-neutral loss of heterozygosity on chromosome 4q24. Compared with patients with monoallelic TET2 mutations（*n*＝147）and those without TET2 mutations（*n*＝1 544）, patients with bi-TET2 carried more gene mutations overall［4（*IQR*: 3, 6）*vs* 4（*IQR*: 2, 5）*vs* 2（*IQR*: 1, 4）, *P*<0.001］and had higher frequencies of mutations in ASXL1（38.5％ *vs* 29.9％ *vs* 20.5％, *P*＝0.001）, SRSF2（28.2％ *vs* 12.9％ *vs* 4.5％, *P*<0.001）, SF3B1（25.6％ *vs* 18.4％ *vs* 11.7％, *P*＝0.004）, EZH2（18.0％ *vs* 7.5％ *vs* 4.5％, *P*＝0.002）, CUX1（12.8％ *vs* 3.4％ *vs* 1.4％, *P*<0.001）, KRAS（10.3％ *vs* 4.3％ *vs* 2.1％, *P*＝0.006）, CBL（10.3％ *vs* 3.4％ *vs* 2.6％, *P*＝0.030）, ZRSR2（10.3％ *vs* 6.1％ *vs* 3.2％, *P*＝0.017）, and CEBPA（7.7％ *vs* 1.4％ *vs* 1.2％, *P*＝0.019）. Patients with bi-TET2 were older at diagnosis［65（*IQR*: 58, 69）*vs* 61（*IQR*: 50, 67）*vs* 55（*IQR*: 44, 64）years, *P*<0.001］and had a higher proportion of normal karyotype（69.2％ *vs* 46.3％ *vs* 44.8％, *P*＝0.024）. Patients with bi-TET2 had a significantly shorter median overall survival（OS）time than patients with monoallelic TET2 mutations and those without TET2 mutations［18.5（95％ *CI*: 13.1–25.0）*vs* 40.6（95％ *CI*: 22.8–60.9）*vs* 54.7（95％ *CI*: 47.1–69.4）months, *P*＝0.009］. Stratified analysis by IPSS-M prognostic risk categories showed that, in the high-risk group（moderate high-risk, high-risk, and very high-risk）, patients with bi-TET2 had a significantly shorter median OS time［12.4（95％ *CI*: 8.4–NA）months］than patients with monoallelic TET2 mutations［19.7（95％ *CI*: 14.9–39.9）months］and those without TET2 mutations［32.0（95％ *CI*: 27.6–37.4）months, *P*<0.001］. In contrast, no significant difference in OS was observed in the low-risk group（very low-risk, low-risk, and moderate low-risk）.

**Conclusion:**

Patients with MDS harboring biallelic TET2 inactivation exhibit distinct clinical and molecular characteristics and in patients with relatively high IPSS-M risk, their prognosis is worse than that of patients with monoallelic TET2 mutations.

骨髓增生异常肿瘤（myelodysplastic neoplasms, MDS），既往亦称骨髓增生异常综合征（myelodysplastic syndromes, MDS），是一组起源于造血干/祖细胞的克隆性疾病，具有高度的生物学和临床异质性[1]–[2]。随着分子遗传学检测技术的广泛应用，分子遗传学异常在MDS的诊断、分型及预后评估中的作用日益凸显，部分特定基因异常已被证明可界定具有独特临床和生物学特征的疾病亚型[3]–[4]。TET2基因突变在MDS患者中较为常见，其可通过影响DNA去甲基化过程干扰造血分化[5]。既往研究提示，TET2单等位基因突变多作为髓系肿瘤发生早期的克隆事件，其临床表型及预后意义在不同研究中报道不一致[6]–[8]。近期研究发现，相较于单等位基因突变，TET2双等位基因失活可能通过基因剂量效应导致更显著的生物学异常，提示其可能构成一类具有相对独立生物学特征的分子亚型[9]。本研究回顾性分析了本中心伴TET2双等位基因失活的MDS患者临床特征及预后，现报道如下。

## 病例与方法

1. 病例资料：本研究为单中心回顾性队列研究。纳入2016年8月至2025年6月于中国医学科学院血液病医院MDS和MPN诊疗中心初诊原发MDS患者1 730例，中位年龄56（*IQR*：46，65）岁，其中男性患者1 088例（62.9％）。患者按世界卫生组织（WHO）2016标准[10]诊断，并根据WHO 2022标准[11]进行重新分型。所有患者临床资料完整，并均签署知情同意书。根据MDS特异性合并疾病指数（MDS-CI）[12]对患者进行合并疾病评估。依据修订的MDS国际预后积分系统（Revised International Prognostic Scoring System, IPSS-R）[13]和分子国际预后评分系统（the Molecular International Prognostic Scoring System, IPSS-M）[14]对患者进行预后分组评估。将IPSS-R极低危、低危、中危合并为IPSS-R相对低危组，将IPSS-R高危、极高危合并为IPSS-R相对高危组。将IPSS-M极低危、低危及较低危组合并为IPSS-M相对低危组，IPSS-M较高危、高危及极高危组合并为IPSS-M相对高危组。

2. 染色体核型分析：采用短期培养法制备染色体标本，经R显带技术进行核型分析，核型描述遵循人类细胞遗传学国际命名体制（ISCN2016）。依据IPSS-R对染色体核型进行预后分组。

3. 靶向二代测序分析：分离患者骨髓单个核细胞提取DNA，对血液肿瘤相关基因进行扩增和富集后测序。测序结果结合CCDS、dbSNP（v138）、1000 Genomes、COSMIC等数据库进行生物信息学分析，筛选致病/可能致病性突变。具体检测流程及数据分析方法参见本中心既往报道[15]–[17]。

4. TET2双等位基因失活定义：根据既往文献报道研究方法[3],[9],[18]，对TET2基因突变患者的等位基因状态进行判定与分组。TET2双等位基因失活（biallelic TET2 inactivation，bi-TET2i）定义为以下任一情况：①同一患者中检测到≥2个TET2基因突变，且其中变异等位基因频率（variant allele frequency，VAF）最高的两个突变的VAF值之和>50％；②检测到单个TET2基因突变，但其VAF值>50％，同时伴有染色体4q24位点（TET2基因座）缺失或拷贝数中性杂合性缺失（copy-neutral loss of heterozygosity，cnLOH）；③通过单核苷酸多态性（single nucleotide polymorphism，SNP）分析证实4q24位点存在单亲二倍体（uniparental disomy，UPD），导致TET2基因突变呈纯合状态。不符合上述标准的TET2基因突变定义为TET2单等位基因突变。未检测到TET2基因突变者定义为无TET2基因突变。

5. 治疗：1 730例患者中可追踪到治疗方案的共1 621例（93.7％），其中382例（23.6％）接受去甲基化±维奈克拉治疗，65例（4.0％）接受联合化疗，459例（28.3％）接受免疫调节/抑制剂±促造血治疗，178例（11.0％）接受促造血±输血治疗，360例（22.2％）接受造血干细胞移植治疗，28例（1.7％）入组临床试验，75例（4.6％）单纯接受中医药治疗，74例（4.6％）选择等待与观察。

6. 随访：采用电话联系、查阅住院或门诊病历的方式对患者进行随访，末次随访时间为2025年10月。所有患者的中位随访时间为26.0（95％ *CI*：23.8～28.3）个月，TET2双等位基因失活组中位随访30.2（95％ *CI*：15.1～45.3）个月，TET2单等位基因突变组中位随访24.6（95％ *CI*：14.6～34.6）个月，无TET2基因突变组中位随访26.2（95％ *CI*：23.8～28.6）个月。主要终点包括总生存（overall survival, OS）和无白血病生存（leukemia-free survival，LFS）期。OS期定义为自确诊MDS起至患者死亡、行造血干细胞移植或末次随访日期。LFS期定义为自确诊MDS起至患者发生白血病转化、死亡、行造血干细胞移植或末次随访日期。

7. 统计学处理：采用R 4.4.2进行统计学分析和绘图。连续变量以中位数（*IQR*）进行描述，组间比较使用Kruskal-Wallis *H*或Mann-Whitney *U*检验。分类变量以例数（百分比）表示，组间比较采用卡方检验或Fisher确切概率法。组内两两比较采用Bonferroni校正法（以*P*<0.017为差异有统计学意义）。使用Kaplan-Meier法进行生存分析并绘制生存曲线，组间比较使用Log-rank检验。*P*<0.05为差异具有统计学意义。

## 结果

一、TET2基因突变及双等位基因失活的特征

1 730例患者中，共有186例（10.8％）患者检出257个TET2基因突变，其中39例（2.3％）符合TET2双等位基因失活的判定标准，其余147例（8.5％）为TET2单等位基因突变。39例TET2双等位基因失活患者中，2例检出染色体4q24位点cnLOH。

39例TET2双等位基因失活患者中，VAF相对较高的TET2基因突变的中位VAF值为43.6％（*IQR*：34.7％～48.1％），多数为移码突变（19个，48.7％），其次为无义突变（11个，28.2％）、错义突变（7个，17.9％）和剪接相关突变（2个，5.1％）。18个突变（46.2％）位于N端非催化区，4个突变（10.3％）位于富半胱氨酸区，12个突变（30.8％）位于双链β-螺旋结构域，5个突变（12.8％）位于N端与C端之间的中间区。VAF相对较低的TET2基因突变的中位VAF值为26.7％（*IQR*：10.8％～37.8％），多数为移码突变（17个，43.5％），其次为无义突变（11个，28.2％）、错义突变（8个，20.5％）和剪接相关突变（2个，5.1％）。

147例TET2单等位基因突变患者中，多数为无义突变（55个，37.1％），其次为移码突变（49个，33.1％）、错义突变（37个，25.0％）和剪接相关突变（7个，4.6％）。TET2基因突变的中位VAF值为21.8％（*IQR*：4.1％～42.9％）。

二、TET2基因突变患者的基因共突变情况

三组患者携带的基因突变总数分布存在显著差异（*P*<0.001，图1），其中TET2双等位基因失活组的基因突变总数为4（*IQR*：3，6）个，TET2单等位基因突变组为4（*IQR*：2，5）个，无TET2基因突变组基因突变总数最低［2（*IQR*：1，4）个］。三组患者的的共突变基因频率见图2。与TET2单等位基因突变患者和无TET2基因突变患者相比，TET2双等位基因失活患者ASXL1（*P*＝0.001）、SRSF2（*P*<0.001）、SF3B1（*P*＝0.004）、EZH2（*P*＝0.002）、CUX1（*P*<0.001）、KRAS（*P*＝0.006）、CBL（*P*＝0.030）、ZRSR2（*P*＝0.017）、CEBPA（*P*＝0.019）基因突变检出率更高。而DNMT3A（*P*＝0.023）基因突变检出率相对其他两组更低。

**图1:**
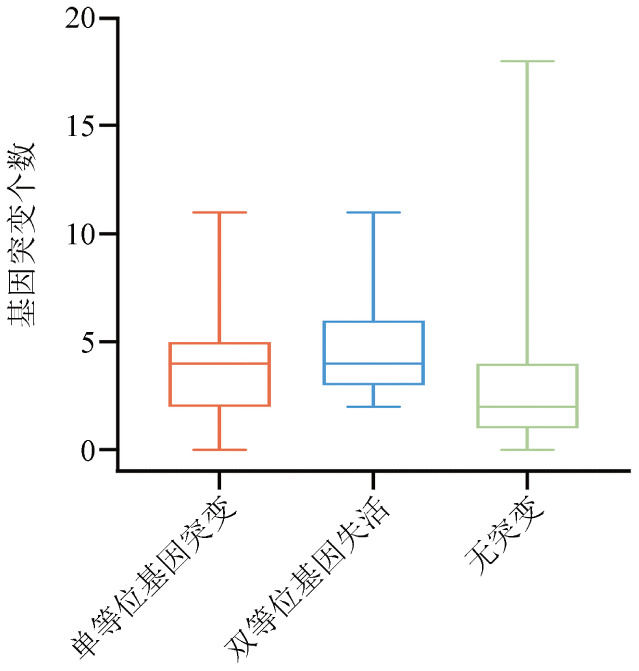
TET2单等位基因突变组（147例）、TET2双等位基因失活组（39例）和无TET2基因突变组（1 544例）骨髓增生异常肿瘤患者的基因突变个数比较（三组整体比较*P*<0.001，TET2双等位基因失活组与TET2单等位基因突变组比较*P*＝0.017；TET2双等位基因失活组与无TET2基因突变组比较*P*<0.001，TET2单等位基因突变组与无TET2基因突变组比较*P*<0.001）

**图2:**
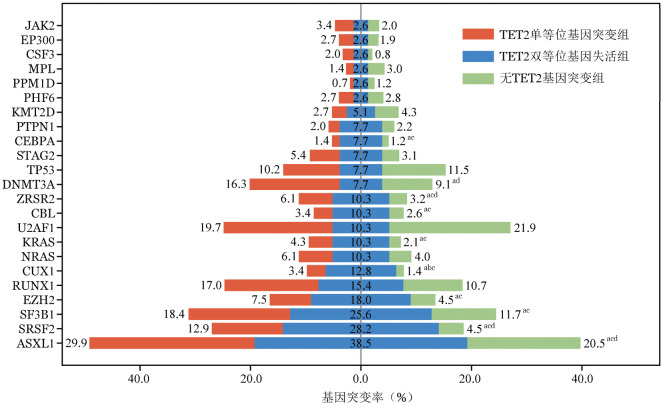
TET2单等位基因突变组（147例）、TET2双等位基因失活组（39例）和无TET2基因突变组（1 544例）骨髓增生异常肿瘤患者共突变基因检出率比较（^a^三组整体比较*P*<0.05；^b^TET2双等位基因失活组与TET2单等位基因突变组比较*P*<0.017；^c^TET2双等位基因失活组与无TET2基因突变组比较*P*<0.017；^d^TET2单等位基因突变组与无TET2基因突变组比较*P*<0.017）

三、TET2双等位基因失活患者的临床特征

TET2双等位基因失活组患者的中位年龄为65（*IQR*：58，69）岁，TET2单等位基因突变组为61（*IQR*：50，67）岁，无TET2基因突变组为55（*IQR*：44，64）岁，三组间年龄分布差异具有统计学意义（*P*<0.001）。三组患者的性别、初诊外周血白细胞计数、中性粒细胞计数、单核细胞计数、血红蛋白水平、血小板计数、WHO2016及2022诊断分型、骨髓原始细胞比例差异均无统计学意义（均*P*>0.05）。细胞遗传学方面，三组染色体核型分布存在差异（*P*＝0.024），其中TET2双等位基因失活组正常核型比例最高（69.2％），高于TET2单等位基因突变组（46.3％）及无TET2基因突变组（44.8％）。IPSS-R及IPSS-M风险分层在三组间未见显著差异（均*P*>0.05）（表1）。

**表1:** 伴TET2双等位基因失活组、TET2单等位基因突变组及无TET2基因突变组骨髓增生异常肿瘤（MDS）患者临床特征比较

临床特征	TET2双等位基因失活 （39例）	TET2单等位基因突变 （147例）	无TET2基因突变组 （1 544例）	*P*值
年龄［岁，*M*（*IQR*）］	65（58, 69）^a^	61（50, 67）^a^	55（44, 64）	<0.001
性别［例（％）］				0.145
男性	28（71.8）	101（68.7）	959（62.1）	
女性	11（28.2）	46（31.3）	585（37.9）	
骨髓原始细胞比例［％, *M*（*IQR*）］	3.0（1.0, 6.5）	2.5（0.5, 7.5）	2.5（1.0, 7.0）	0.391
骨髓环铁细胞比例［％, *M*（*IQR*）］	0（0, 10）	0（0, 2）	0（0, 2）	0.172
外周血细胞计数				
WBC［×10^9^/L, *M*（*IQR*）］	2.87（1.83, 4.33）	2.77（2.00, 3.64）	2.59（1.81, 3.69）	0.422
ANC［×10^9^/L, *M*（*IQR*）］	1.30（0.73, 2.92）	1.21（0.68, 2.07）	1.08（0.62, 1.96）	0.188
Monocytes［×10^9^/L, *M*（*IQR*）］	0.32（0.14, 0.66）	0.22（0.11, 0.40）	0.20（0.11, 0.43）	0.120
HGB［g/L, *M*（*IQR*）］	79（62, 96）	79（66, 100）	80（66, 97）	0.637
PLT［×10^9^/L, *M*（*IQR*）］	61（28, 186）	68（32, 140）	64（34, 128）	0.807
WHO 2016诊断分型［例（％）］				0.070
MDS-SLD/MLD	15（38.5）	60（41.1）	691（45.0）	
MDS-RS-SLD/MLD	8（20.5）	16（10.9）	136（8.9）	
MDS-EB1/2	14（35.9）	63（43.1）	623（40.6）	
MDS-del（5q）	0（0）	2（1.4）	23（1.5）	
MDS-U	2（5.1）	5（3.4）	62（4.0）	
WHO 2022诊断分型［例（％）］				0.415
MDS-IB1/IB2	11（28.9）	49（33.3）	462（31.2）	
MDS-LB	15（39.5）	52（35.4）	570（38.5）	
MDS-biTP53	2（5.3）	10（6.8）	109（7.4）	
MDS-SF3B1	8（21.1）	18（12.2）	140（9.4）	
MDS-5q	0（0）	1（0.7）	22（1.5）	
MDS-h	0（0）	7（4.8）	120（8.1）	
MDS-f	2（5.3）	7（4.8）	59（4.0）	
染色体核型［例（％）］				
正常核型	27（69.2）^b^	68（46.3）	691（44.8）	0.024
复杂核型	3（7.7）	16（10.9）	227（14.7）	0.217
del（5q）	0（0）	4（2.7）	100（6.5）	0.041
+8	7（17.9）	18（12.2）	176（11.4）	0.484
−7	2（5.1）	8（5.4）	112（7.3）	0.739
MDS-CI得分［*M*（*IQR*）］	0（0, 0）	0（0, 1）	0（0, 0）	0.324
IPSS-R分组［例（％）］				0.498
极低危	1（2.7）	8（5.9）	62（4.3）	
低危	12（32.4）	40（29.4）	365（25.5）	
中危	14（37.8）	38（27.9）	435（30.4）	
高危	8（21.6）	26（19.1）	330（23.1）	
极高危	2（5.4）	24（17.6）	239（16.7）	
IPSS-R合并分组［例（％）］				0.243
相对低危	27（73.0）	86（63.2）	862（60.2）	
相对高危	10（27.0）	50（36.8）	569（39.8）	
IPSS-M分组［例（％）］				0.520
极低危	0（0）	3（2.2）	37（2.7）	
低危	10（27.0）	29（21.3）	250（18.1）	
较低危	6（16.2）	23（16.9）	195（14.1）	
较高危	2（5.4）	15（11.0）	224（16.2）	
高危	10（27.0）	30（22.1）	342（24.7）	
极高危	9（24.3）	36（26.5）	336（24.3）	
IPSS-M合并分组［例（％）］				0.260
相对低危	16（43.2）	55（40.4）	482（34.8）	
相对高危	21（56.8）	81（59.6）	902（65.2）	

**注** Monocytes：单核细胞；MDS-SLD/MLD：MDS伴单系/多系发育异常；MDS-RS：MDS伴环形铁粒幼红细胞；MDS-EB：MDS伴原始细胞增多；MDS-del（5q）：MDS伴单纯del（5q）；MDS-U：MDS不能分类；MDS-IB：MDS伴原始细胞增多；MDS-biTP53：MDS伴TP53双等位基因失活；MDS-SF3B1：MDS伴低原始细胞和SF3B1突变；MDS-h：低增生MDS；MDS-f：MDS伴纤维化；MDS-CI：MDS特异性合并疾病指数；IPSS-R：修订版国际预后评分系统，IPSS-R极低危、低危、中危为相对低危组，IPSS-R高危、极高危为相对高危组；IPSS-M：分子国际预后评分系统，IPSS-M极低危、低危及较低危为相对低危组，IPSS-M较高危、高危及极高危为相对高危组。^a^与无TET2基因突变组相比，*P*<0.001；^b^与无TET2基因突变组相比，*P*<0.01

四、TET2双等位基因失活患者的预后分析

截至随访终点，共530例（30.6％）患者死亡，其中TET2双等位基因失活组死亡18例（46.1％），TET2单等位基因突变组死亡50例（34.0％），无TET2基因突变组死亡462例（29.9％）。TET2双等位基因失活组患者中位OS期［18.5（95％ *CI*：13.1～25.0）个月］较TET2单等位基因突变组［40.6（95％ *CI*：22.8～60.9）个月］和无TET2基因突变组［54.7（95％ *CI*：47.1～69.4）个月］显著缩短，差异有统计学意义（*P*＝0.009，图3A）。

**图3:**
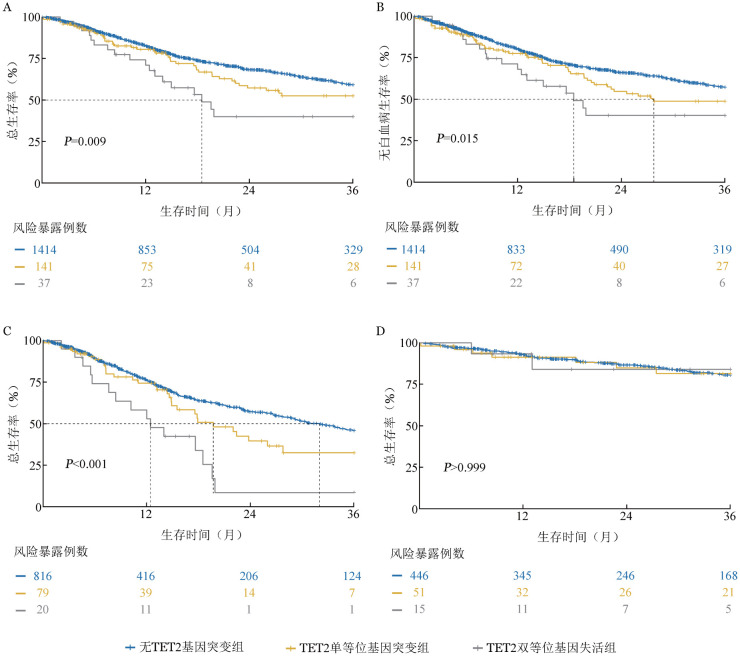
伴TET2双等位基因失活、TET2单等位基因突变、无TET2基因突变的骨髓增生异常肿瘤患者总生存及无白血病生存曲线 **A** 三组患者总生存比较；**B** 三组患者无白血病生存比较；**C** IPSS-M相对高危亚组总生存比较；**D** IPSS-M相对低危亚组总生存比较 **注** IPSS-M：分子国际预后评分系统，极低危、低危及较低危为相对低危组，较高危、高危及极高危为相对高危组

TET2双等位基因失活组患者中位LFS期［18.5（95％ *CI*：13.07～NA）个月］较TET2单等位基因突变组［27.8（95％ *CI*：20.9～48.8）个月］和无TET2基因突变组［50.6（95％ *CI*：43.3～63.3）个月］显著缩短，差异有统计学意义（*P*＝0.015，图3B）。

基于IPSS-M预后风险分层进行亚组分析发现，在IPSS-M相对高危组中，三组患者的中位OS期差异具有统计学意义（*P*<0.001）。TET2双等位基因失活患者中位OS期仅为12.4（95％ *CI*：8.4～NA）个月，显著短于无TET2基因突变患者［32.0（95％ *CI*：27.6～37.4）个月，*P*<0.001］和TET2单等位基因突变患者［19.7（95％ *CI*：14.9～39.9）个月，*P*<0.001］（图3C）。而在IPSS-M相对低危组中，三组间OS差异无统计学意义（*P*>0.999，图3D）。

## 讨论

本研究系统分析了TET2双等位基因失活患者的分子特征、临床特征及预后意义。TET2基因编码一种依赖α-酮戊二酸和二价铁离子的双加氧酶，可催化DNA中的5-甲基胞嘧啶（5mC）氧化为5-羟甲基胞嘧啶（5hmC），从而启动DNA去甲基化过程[19]。这一表观遗传修饰对于维持造血干细胞的自我更新与分化平衡至关重要。TET2功能缺失会导致高甲基化状态，阻碍髓系祖细胞的正常分化成熟，并促进其向单核细胞系分化。既往的TET2基因突变相关研究多将其作为整体分析，而本研究进一步区分了TET2双等位基因失活与单等位基因突变状态，发现二者在分子共突变谱、部分临床特征及预后影响方面存在差异，提示TET2的等位基因状态在MDS风险评估中具有补充价值。

本研究TET2基因突变的检出率为10.8％，其中2.3％的患者符合双等位基因失活判定标准。既往Awada等[9]在以髓系肿瘤为主的大型队列研究中报道，TET2突变的总体检出率为19.1％，其中7.8％的患者存在双等位基因失活，其发生率高于本研究。两者差异可能与研究对象构成不同有关，Awada等[9]的研究中纳入了慢性粒-单核细胞白血病（CMML）患者，而既往研究显示CMML患者中TET2基因突变和双等位基因失活的发生率分别可达64.7％和55.4％[20]。本研究仅纳入原发MDS患者，可能导致TET2基因突变及双等位基因失活的检出率相对较低。

与既往报道一致[5]–[6],[21]，本研究中TET2双等位基因失活和TET2单等位基因突变均以移码突变和无义突变为主，符合TET2作为抑癌基因、以功能缺失性突变为主要致病形式的生物学特征。TET2双等位基因失活多发生于多突变背景，常伴随多种其他基因异常，其中以表观遗传调控相关基因（如ASXL1）、剪接因子基因（尤其是SRSF2）以及RAS通路相关基因（KRAS/NRAS等）较为常见[3],[7],[9]。与此一致，本研究观察到TET2双等位基因失活在MDS患者中常与上述突变共同出现。与部分既往研究不同的是，本研究中SF3B1、EZH2、CUX1、CBL、ZRSR2及CEBPA突变在TET2双等位基因失活患者中亦相对多见，其差异可能与研究对象疾病谱不同、测序覆盖范围及人群遗传背景差异有关。

在临床特征方面，本研究发现TET2双等位基因失活患者的发病年龄显著高于TET2单等位基因突变及无TET2基因突变患者，且TET2单等位基因突变患者的发病年龄亦高于无TET2基因突变患者。该现象可能与衰老过程中TET2突变在克隆性造血中逐渐积累，并在长期演化过程中进一步获得继发性亚克隆性打击有关[22]–[23]。考虑到各组患者年龄差异较大，而老年人群往往合并症负担相对更重，对预后有一定影响，因此本研究进一步采用MDS-CI对患者的基线合并症负担进行评估。结果显示三组患者的MDS-CI评分分布相近，提示TET2双等位基因失活患者虽然年龄更大，但未伴随更重的总体合并症负担，三组患者之间临床特征和预后的差异更可能与TET2等位基因受损程度及其分子背景相关。既往研究显示，TET2双等位基因失活的患者常伴随单核细胞增多及髓系发育异常[24]–[25]。Bernard等[3]的研究结果表明，TET2双等位基因失活患者贫血程度较轻，血小板减少更明显，且骨髓原始细胞比例更高。然而，在本研究中未观察到上述外周血及骨髓特征的显著差异，其可能原因是研究人群构成不同，Bernard等[3]的研究纳入了MDS、MDS/MPN及AML患者，而本研究仅分析原发MDS人群；另外，本研究TET2双等位基因失活患者数量相对有限，可能影响部分临床特征差异的统计学检出。

在细胞遗传学特征方面，既往多项研究证实TET2突变更常见于正常核型的髓系肿瘤患者[26]–[27]。Awada等[9]对1 045例髓系肿瘤患者的分析进一步显示，TET2双等位基因失活患者更常表现为正常染色体核型。本研究结果与上述研究一致，同样观察到TET2双等位基因失活患者的正常核型比例显著高于TET2单等位基因突变及无TET2突变患者。

既往关于TET2基因突变与MDS预后关系的研究结论并不一致。Kosmider等[6]认为TET2基因突变是MDS的独立有利预后因素，而Alexander等[7]的研究未发现TET2基因突变对MDS患者的生存期有显著影响。但本研究进一步区分TET2等位基因状态后明确发现，在IPSS-M相对高危人群中，TET2双等位基因失活患者的OS期明显缩短，而在IPSS-M相对低危人群中未见显著差异。TET2功能受损可导致5hmC水平降低[19]，而5hmC的下降与恶性表型和不良生存预后相关[28]。当两个等位基因均受损时，其功能缺失程度可能更为明显。Awada等[9]提出部分病例可能存在残余功能或代偿效应，通过过乙酰化等机制部分维持残余功能，从而缓冲其临床不良影响。而在本研究中，在IPSS-M高危背景下，该潜在补偿机制可能不足以抵消TET2双等位基因失活带来的不良影响，OS期显著缩短。

针对TET2双等位基因失活MDS患者的不良预后，探索有效的治疗策略尤为重要。异基因造血干细胞移植（allo-HSCT）是目前MDS唯一的根治手段，尽管既往研究提示TET2基因突变与MDS患者移植后OS较差相关[29]，但未进一步区分等位基因状态对移植结局的影响。受限于本研究中TET2双等位基因失活患者队列人数相对较少，且接受移植的病例数有限，目前尚不足以评估allo-HSCT相对于非移植治疗是否能为该亚组带来明确的生存获益。鉴于双等位基因失活患者在IPSS-M相对高危组中的生存表现较差，allo-HSCT能否作为改善该亚组结局的有效策略，仍需在大样本队列中进一步验证。

综上所述，本研究表明TET2双等位基因失活患者的年龄更大，且更常表现为正常染色体核型。与TET2单等位基因突变及无TET2基因突变患者相比，TET2双等位基因失活患者的OS和LFS期均缩短，其不良预后效应主要体现在IPSS-M相对高危人群中，提示TET2双等位基因失活在IPSS-M相对高危MDS患者中有一定的预后指示价值。鉴于IPSS-M分子预后系统未将TET2的等位基因状态纳入评分体系，因此，在IPSS-M评估基础上进一步关注TET2等位基因状态，可能有助于更精细地识别部分高危患者。

本研究的局限性在于：①为单中心、回顾性分析，存在一定选择偏倚；②TET2双等位基因失活患者数量相对有限，部分亚组分析的统计效能不足；③双等位基因失活的判定依赖于VAF及拷贝数，不同检测平台和分析方法可能有所影响。未来仍需在更大样本、多中心、前瞻性队列中进一步验证TET2双等位基因失活的临床和预后意义。

## References

[1] Cazzola M (2020). Myelodysplastic Syndromes[J]. N Engl J Med.

[2] 肖 志坚 (2015). 骨髓增生异常综合征的精确诊断[J]. 中华血液学杂志.

[3] Bernard E, Hasserjian RP, Greenberg PL (2024). Molecular taxonomy of myelodysplastic syndromes and its clinical implications[J]. Blood.

[4] 肖 志坚 (2025). 分子遗传学在骨髓增生异常肿瘤诊治中的应用:现况与问题[J]. 国际输血及血液学杂志.

[5] Delhommeau F, Dupont S, Della Valle V (2009). Mutation in TET2 in myeloid cancers[J]. N Engl J Med.

[6] Kosmider O, Gelsi-Boyer V, Cheok M (2009). Groupe Francophone des Myélodysplasies. TET2 mutation is an independent favorable prognostic factor in myelodysplastic syndromes (MDSs)[J]. Blood.

[7] Smith AE, Mohamedali AM, Kulasekararaj A (2010). Next-generation sequencing of the TET2 gene in 355 MDS and CMML patients reveals low-abundance mutant clones with early origins, but indicates no definite prognostic value[J]. Blood.

[8] Kim M, Yahng SA, Kwon A (2015). Mutation in TET2 or TP53 predicts poor survival in patients with myelodysplastic syndrome receiving hypomethylating treatment or stem cell transplantation[J]. Bone Marrow Transplant.

[9] Awada H, Nagata Y, Goyal A (2019). Invariant phenotype and molecular association of biallelic TET2 mutant myeloid neoplasia[J]. Blood Adv.

[10] Arber DA, Orazi A, Hasserjian R (2016). The 2016 revision to the World Health Organization classification of myeloid neoplasms and acute leukemia[J]. Blood.

[11] Alaggio R, Amador C, Anagnostopoulos I (2022). The 5th edition of the World Health Organization Classification of Haematolymphoid Tumours: Lymphoid Neoplasms[J]. Leukemia.

[12] Della Porta MG, Malcovati L, Strupp C (2011). Risk stratification based on both disease status and extra-hematologic comorbidities in patients with myelodysplastic syndrome[J]. Haematologica.

[13] Greenberg PL, Tuechler H, Schanz J (2012). Revised international prognostic scoring system for myelodysplastic syndromes[J]. Blood.

[14] Bernard E, Tuechler H, Greenberg PL (2022). Molecular international prognostic scoring system for myelodysplastic syndromes[J]. NEJM Evid.

[15] Li B, Liu J, Jia Y (2018). Clinical features and biological implications of different U2AF1 mutation types in myelodysplastic syndromes[J]. Genes Chromosomes Cancer.

[16] Zhang Y, Wu J, Qin T (2022). Comparison of the revised 4th (2016) and 5th (2022) editions of the World Health Organization classification of myelodysplastic neoplasms[J]. Leukemia.

[17] 李 冰, 王 静雅, 刘 晋琴 (2017). 靶向测序检测511例骨髓增生异常综合征患者基因突变[J]. 中华血液学杂志.

[18] Bernard E, Nannya Y, Hasserjian RP (2020). Implications of TP53 allelic state for genome stability, clinical presentation and outcomes in myelodysplastic syndromes[J]. Nat Med.

[19] Ko M, Huang Y, Jankowska AM (2010). Impaired hydroxylation of 5-methylcytosine in myeloid cancers with mutant TET2[J]. Nature.

[20] Kynning MK, Westerberg E, Forsell L (2026). Comorbidities and mutations including single- and multihit TET2 mutations in relation to outcome in chronic myelomonocytic leukaemia-A population-based study[J]. Br J Haematol.

[21] Langemeijer SM, Kuiper RP, Berends M (2009). Acquired mutations in TET2 are common in myelodysplastic syndromes[J]. Nat Genet.

[22] Xie M, Lu C, Wang J (2014). Age-related mutations associated with clonal hematopoietic expansion and malignancies[J]. Nat Med.

[23] Busque L, Patel JP, Figueroa ME (2012). Recurrent somatic TET2 mutations in normal elderly individuals with clonal hematopoiesis[J]. Nat Genet.

[24] Tefferi A, Lim KH, Abdel-Wahab O (2009). Detection of mutant TET2 in myeloid malignancies other than myeloproliferative neoplasms: CMML, MDS, MDS/MPN and AML[J]. Leukemia.

[25] Santos IM, Franzon CM, Koga AH (2012). Laboratory diagnosis of chronic myelomonocytic leukemia and progression to acute leukemia in association with chronic lymphocytic leukemia: morphological features and immunophenotypic profile[J]. Rev Bras Hematol Hemoter.

[26] Hirsch CM, Nazha A, Kneen K (2018). Consequences of mutant TET2 on clonality and subclonal hierarchy[J]. Leukemia.

[27] Bejar R, Lord A, Stevenson K (2014). TET2 mutations predict response to hypomethylating agents in myelodysplastic syndrome patients[J]. Blood.

[28] Liu X, Zhang G, Yi Y (2013). Decreased 5-hydroxymethylcytosine levels are associated with TET2 mutation and unfavorable overall survival in myelodysplastic syndromes[J]. Leuk Lymphoma.

[29] Bejar R, Stevenson KE, Caughey B (2014). Somatic mutations predict poor outcome in patients with myelodysplastic syndrome after hematopoietic stem-cell transplantation[J]. J Clin Oncol.

